# Bayesian Noise Modelling for State Estimation of the Spread of COVID-19 in Saudi Arabia with Extended Kalman Filters

**DOI:** 10.3390/s23104734

**Published:** 2023-05-13

**Authors:** Lamia Alyami, Deepak Kumar Panda, Saptarshi Das

**Affiliations:** 1 Centre for Environmental Mathematics, Faculty of Environment, Science and Economy, University of Exeter, Penryn Campus, Penryn TR10 9FE, UK; la424@exeter.ac.uk (L.A.); dp457@exeter.ac.uk (D.K.P.); 2 Department of Mathematics, College of Science, Najran University, Najran 11001, Saudi Arabia; 3 Institute for Data Science and Artificial Intelligence, University of Exeter, Exeter EX4 4QE, UK

**Keywords:** Bayesian model selection, nested sampling, skew-normal distributions, Bayesian evidence, extended Kalman filter (EKF)

## Abstract

The epistemic uncertainty in coronavirus disease (COVID-19) model-based predictions using complex noisy data greatly affects the accuracy of pandemic trend and state estimations. Quantifying the uncertainty of COVID-19 trends caused by different unobserved hidden variables is needed to evaluate the accuracy of the predictions for complex compartmental epidemiological models. A new approach for estimating the measurement noise covariance from real COVID-19 pandemic data has been presented based on the marginal likelihood (Bayesian evidence) for Bayesian model selection of the stochastic part of the Extended Kalman filter (EKF), with a sixth-order nonlinear epidemic model, known as the SEIQRD (Susceptible–Exposed–Infected–Quarantined–Recovered–Dead) compartmental model. This study presents a method for testing the noise covariance in cases of dependence or independence between the infected and death errors, to better understand their impact on the predictive accuracy and reliability of EKF statistical models. The proposed approach is able to reduce the error in the quantity of interest compared to the arbitrarily chosen values in the EKF estimation.

## 1. Introduction

During the COVID-19 pandemic, there have been various mathematical modelling approaches used to forecast the behaviour of the pandemic, including different stochastic and deterministic compartmental models, in order to achieve greater predictive accuracy. Despite all these efforts, there is a large uncertainty associated with the nature of COVID-19’s spreading dynamics. This uncertainty could be due to modelling errors; hidden or unknown factors; incomplete, missing or delayed reporting of data; and other unknown disturbances. In our previous work [1], we fitted a deterministic model called SEIQRD (Susceptible–Exposed– Infected–Quarantined–Recovered–Dead) using the COVID-19 data from Saudi Arabia to predict the evolution of the number of active cases and fatal cases. We used the nested sampling algorithm [2] to fit the epidemiological model’s parameters to obtain the multivariate posterior mean of the parameters for simulation. However, we observed a difference between the mean of the deterministic model and the measured data. This difference lies within the modelled uncertainty or error bounds derived from the posterior samples. This has motivated us to further focus on modelling the noise (i.e., the aleatoric uncertainty) to fit this difference or the noise to the best possible probability distribution, which may not necessarily be symmetric in nature. Therefore, in this paper, we aim to identify the distribution that best describes the noise between a smooth deterministic model and the real-world fluctuations in COVID-19 data. To achieve this, we have fitted the parameters of the noise or residuals (i.e., the difference between real data and deterministic SEIQRD model output), which may help to frame a Kalman filtering (KF) problem with greater precision.

Previously, in [1], for the state estimation results using the EKF with pre-assumed system error covariance (Ξ) and measurement error covariance (Ω) matrices, the noise distributions were empirical since the accurate values of the noise covariances are generally unknown in many applications. Several methods have been proposed to solve the issue of estimating noise covariances, such as using the least-squares methods, as described in [3,4,5]. Hence, we aim to address this gap from a different perspective by fitting the parameters of the noise distribution to obtain an accurate measurement of the covariance error (Ω) to be used in the EKF state estimation algorithm. This mainly aims to characterize the error of COVID-19 data to better describe the aleatoric uncertainty in addition to the previously tackled epistemic uncertainty and their propagation in state estimation using a nonlinear compartmental pandemic model, called SEIQRD with reinfection. Subsequently, we have proposed a method to resolve an open scientific question from the stochastic data modelling perspective; i.e., within Kalman filtering framework, can we assume that the data-fitting exercise adapts to normality assumptions, or is it strictly applicable to the normal distribution only? We also investigate the consequences of these assumptions of normality and correlation between errors in different variables or using skewed distributions with correlations, etc., in the Kalman filtering framework. In order to address these open questions, we will consider Gaussian and non-Gaussian distributions to represent the errors and fit the residuals between the fitted deterministic model response and real COVID-19 spread data in Saudi Arabia, using generalized Bayesian inference under the Bayesian model selection framework. For linear systems with non-Gaussian noise, the KF remains an optimal state estimator, as described in [6]. This refutes the claims made in much of the published literature, indicating that the KF is not optimal unless the noise is assumed to be Gaussian. Furthermore, the KF in a Gaussian approximation framework only estimates the mean and covariance of the states, which may neglect other valuable information of the underlying distributions, as discussed in [7]. Since many real-world state estimation problems exhibit non-Gaussian patterns, especially when the data are highly skewed or heavy-tailed, it is necessary to consider asymmetric distributions instead of assuming normality.

In recent years, there has been a great deal of interest in skewed distributions and their properties, as demonstrated by [8], who discussed these distributions in detail along with their various applications. Skewed distributions may be necessary to better describe the behaviour of COVID-19 modelling errors in the presence of asymmetric measurement noise. Kalman filtering has also been used in a few studies to estimate the COVID-19 outbreak. Zhu et al. [9] considered a stochastic SEIR model based on EKF to analyse the spread of COVID-19 and quantify the uncertainty with good accuracy as compared to the deterministic model. The ensemble Kalman filter (EnKF) in [10] has been used to estimate the time-varying model parameters with good performance close to the reported data. Developing the EKF to analyse the dynamics of COVID-19 based on maximum likelihood estimation (MLE) for the model parameters has been presented in [11]. Tracking the reproduction number ($R_{0}$) with confidence bounds based on the KF has been introduced in [12]. In addition, some literature has compared the performance of COVID-19 prediction with different types of KFs, e.g., fractional-order EKF with an SEIR model [13], switching KF with time-series models [14], cubature KF with the SEIRRPV model (Susceptible–Exposed–Infected–Recovered from exposure–Recovered from infection–Passed away–Vaccinated) in [15] and Quadratic KF with SEIR/ARIMA (AutoRegressive Integrated Moving Average) models [16]. The novelty of this work as opposed to the existing Kalman filtering works on COVID-19 data is that it reports a thorough benchmarking of the state estimation performances using four alternative formations of the EKF with and without correlated noise and skewed distribution based on the residual of the deterministic model and real data. We do not use the traditional COVID-19 data-driven ARMA family of time series models or other machine learning methods such as neural networks for pandemic modelling since they are mostly black-box models which are difficult to interpret. In contrast, the simple nonlinear deterministic compartmental model is well-studied in the context of epidemic modelling in different scales, and each of the measured or estimated states have meaningful interpretation as opposed to black-box machine learning models. Although recurrent neural networks (RNNs) and other non-parametric time-domain models have been used for the prediction of COVID-19 data, state estimation using compartmental models is a different research direction which has not been addressed in most the contemporary machine learning applications for COVID-19. One of the key motivations here is to estimate the hidden state variables of the compartmental model such as the susceptible, exposed, quarantined, and recovered populations, which is not possible with traditional time-series models as opposed to differential equation-based mechanistic models.

The rest of this paper is organized as follows. In Section 2, we provide a brief review of the KF prediction and the deterministic/stochastic model. In Section 3, the method is outlined. The investigation and the discussion of model comparison results are presented in Section 4, and Section 5 concludes the paper with future research directions.

## 2. Kalman Filtering and the Pandemic Model

We recall the state space model equations without giving the detailed derivation of the EKF algorithm as follows:

(1) $$\begin{array}{cc} X_{t+1} & =f\left(X_{t}\right)+\xi_{t},\;\mathrm{System}\;\mathrm{equation},\\ y_{t} & =HX_{t}+\omega_{t},\;\mathrm{Measurement}\;\mathrm{equation}, \end{array}$$

**Figure 1:**
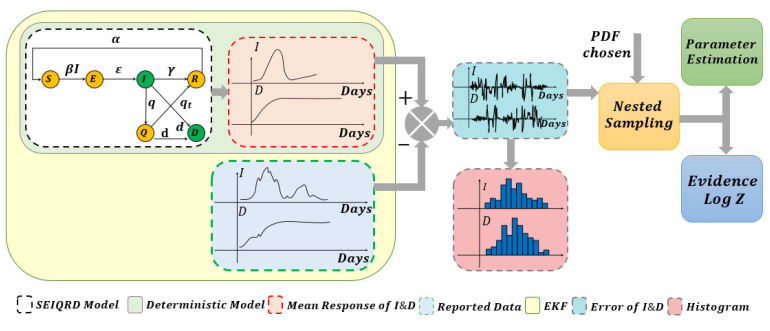
COVID-19 data analysis pipeline with deterministic and stochastic compartments.

**Figure 2:**
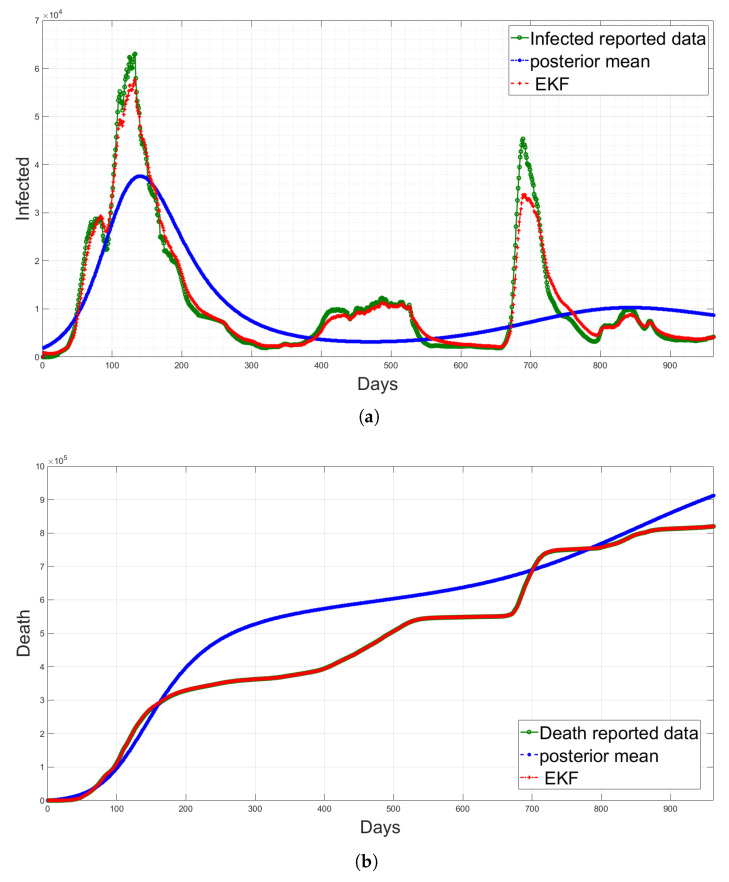
The estimation results of the active cases and death cases using the deterministic model and the EKF. (**a**) Comparison of active cases between the reported data, posterior mean and the proposed EKF; (**b**) Comparison of death cases between the reported data, posterior mean and the proposed EKF.

Here, *f* is the nonlinear function; $\xi_{t}$ is the system noise which is assumed to be Gaussian with a zero mean and covariance matrix Ξ; $y_{t}$ is the measurement sequences for the observed data; *H* is the measurement matrix, with measurement noise $\omega_{t}$ which is Gaussian-distributed with a zero mean and covariance matrix Ω. The deterministic SEIQRD model in [1] divides the population into six classes: susceptible *S*(*t*), exposed *E*(*t*), infected *I*(*t*), quarantined *Q*(*t*), recovered *R*(*t*) and dead *D*(*t*). The compartments were modelled using the system of nonlinear differential equations, as illustrated in the first block in Figure 1. The EKF estimation based on the deterministic SEIQRD model for the active cases and death cases is updated with the new data in this study, as presented in Figure 2. The data are collected from the Saudi Health Ministry available online [17] with daily measurements from 15 March 2020 to 19 October 2022. The variations between the posterior mean (the output of SEIQRD model) and reported data are clear in both observed cases, as shown for active cases in Figure 2a and for death cases in Figure 2b, along with the EKF estimation, which is very close to the reported data.

Now, let us convert this model from the deterministic case to a stochastic case by adding the residuals between the mean posterior response of states *I* and *D*. This will help to capture the randomness in the dynamics of disease spreading model that have been studied in, e.g., [18,19,20] in order to develop more realistic models matching with the real pandemic data. Then, the deterministic SEIQRD system becomes a stochastic model after adding the noise terms as follows:

(2) $$\begin{array}{cc} \frac{dS}{dt} & =-\beta IS+\alpha R,\\ \frac{dE}{dt} & =\beta IS-\epsilon E,\\ \frac{dI}{dt} & =\epsilon E-\gamma I-qI-dI+w_{I},\\ \frac{dQ}{dt} & =qI-q_{t}Q-dQ,\\ \frac{dR}{dt} & =\gamma I+q_{t}Q-\alpha R,\\ \frac{dD}{dt} & =dI+dQ+w_{D}. \end{array}$$

The unknown model parameters {β, γ, ϵ, *q*, $q_{t}$, α and *d*} are non-negative and defined as the infection rate, recovery rate, incubation rate, quarantine rate, quarantine period, reinfection rate and death rate, respectively. Additionally, $w_{I}$ and $w_{D}$ are the noise terms that we investigated in this work which are the residuals between the reported data and the fitted deterministic model reported in our earlier work [1]. We can now express the infected and death residuals, i.e., $w_{I}$ and $w_{D}$ as:

(3) $$w_{I}=y_{I}-{\hat{y}}_{I},\;w_{D}=y_{D}-{\hat{y}}_{D}.$$

We take into account that $w_{I}$ and $w_{D}$ may be independent or may also be correlated while aiming to estimate their trends with the EKF using different noise distributions. Writing these components in a vector format yields:

(4) $$W={ [\begin{array}{cc} w_{I} & w_{D} \end{array}]}^{T}.$$

Next, as shown in Figure 1, $y_{I}$ and $y_{D}$ present the binned frequencies of the error signals since the errors are first binned by turning them into a histogram and the differences in the bins are compared using the new likelihood function. Here, $\hat{y}$ is the binned version of the response of the deterministic SEIQRD model. We examine the residuals *W* with two different distributions. Firstly, we examine a multivariate normal (MVN) distribution in two cases with correlation and without correlation between the parameters in the covariance matrix. Secondly, we examine the multivariate skew-normal (MSN) distribution in two cases with correlation and without correlation between the parameters in the covariance matrix. The dependence between the two components of the error is modelled by the matrix Σ.

### Multivariate Distributions

Now, consider *W*∼MVN(0,Σ) with zero mean vector and covariance matrix Σ with strictly positive diagonal elements as follows:

(5) $$\Sigma = [\begin{array}{cc} {\hat{\sigma }}_{1} & {\hat{\sigma }}_{12}\\ {\hat{\sigma }}_{21} & {\hat{\sigma }}_{2} \end{array}].$$

The likelihood of the MVN distribution can be written as:

(6) $$L\left(\theta \right)={\left(2\pi \right)}^{-n/2}{\left|\Sigma \right|}^{-1/2}\exp\left\{-\frac{1}{2}\prod_{i=1}^{n}{\left(y_{j}-{\hat{y}}_{j}\left(\theta \right)\right)}^{T}\Sigma^{-1}\left(y_{j}-{\hat{y}}_{j}\left(\theta \right)\right)\right\},$$

where n= 32 is the number of bins for the residual data which is the square root of the number of samples chosen as a standard approach for selecting the number of bins. Then, these differences in the bin count are compared in the likelihood function as shown in Equation (6). Taking the log-likelihood function $\ell \left(\theta \right)$ for Equation (6) where individual bins of the histogram are summed up yields:

(7) $$\ell \left(\theta \right)=-\frac{n}{2}\log\left(2\pi \right)-\frac{1}{2}\log\left|\Sigma \right|-\frac{1}{2}\left\{\sum_{j=1}^{n}{\left(y_{j}-{\hat{y}}_{j}\left(\theta \right)\right)}^{T}\Sigma^{-1}\left(y_{j}-{\hat{y}}_{j}\left(\theta \right)\right)\right\},$$

where $\theta =\left({\hat{\sigma }}_{1},{\hat{\sigma }}_{2},{\hat{\sigma }}_{12}\right)$.

The second distribution that we fit the residuals on is the MSN distribution proposed in [21]. The *n*-dimensional random vector *y*-distributed MSN distribution with skewness vector α has a probability density function (pdf) given by:

(8) $$f\left(y\right)=2\phi_{n}\left(y\right)\Phi \left(\alpha^{T}y\right),\;y\in R^{n},\;\alpha \in R^{n},$$

where ϕ(·) and Φ(·) are the pdf and the standard normal cumulative distribution function (cdf) of the *n*-dimensional standard normal distribution N(0,I), respectively. When α= 0, the Equation (8) reduces to $N_{n}\left(0,I_{n}\right)$.

Now consider *W*∼MSN(0,Σ,α) with zero mean vector, covariance matrix Σ and skewness vector α and following [22]. The log-likelihood function ℓ(θ) of the Equation (8) can be written as:

(9) $$\begin{array}{cc} \ell (\theta ) & =-\frac{n}{2}\log\left|\Sigma \right|-\frac{n}{2}\mathrm{tr}\left(\Sigma^{-1}V\right)+\sum_{j}^{n}\log\left[\Phi \left(\alpha^{T}\Sigma^{\frac{-1}{2}}(y_{j}-{\hat{y}}_{j}\left(\theta )\right)\right)\right], \end{array}$$

where

(10) $$V=n^{-1}\sum_{j}^{n}\left(y_{j}-{\hat{y}}_{j}\left(\theta \right)\right){\left(y_{i}-{\hat{y}}_{j}\left(\theta \right)\right)}^{T}.$$

The components of the skewness vector α are expressed as:

(11) $$\alpha ={ [\begin{array}{cc} {\hat{\alpha }}_{1} & {\hat{\alpha }}_{2} \end{array}]}^{T}$$

and $\theta =({\hat{\sigma }}_{1},{\hat{\sigma }}_{2},{\hat{\sigma }}_{12},{\hat{\alpha }}_{1},{\hat{\alpha }}_{2})$.

## 3. Methodology for Model Parameters and Uncertainty Estimation

In Bayesian inference methods, the Bayesian evidence or marginal likelihood (Z) is commonly used for model selection and hypothesis testing, as discussed in various studies, e.g., [23,24,25]. A recent survey [26] extensively explored the computation of marginal likelihood using alternative methods. Many methods have been suggested to compute the marginal likelihood and normalizing constants, as summarized in [27,28,29,30]. Among these methods, we used the nested sampling algorithm proposed in [2] due to its simplicity and efficiency in approximating the marginal likelihood for even large-scale Bayesian inference problems, as shown by [31], in comparison to other methods. The nested sampling and efficient Bayesian inference algorithms have been successfully applied in various fields, including cosmology [32,33], epidemiology [1,34], spatio-temporal inference problems in geophysics [35], material science [36] and other such fields where Bayesian inferences are traditionally applied to fit data with mechanistic models.

Nested sampling is considered to be a generic and efficient Bayesian inference engine that provides the posterior samples for the unknown parameters as a by-product while calculating the marginal likelihood or Bayesian evidence Z. Nested sampling aims to integrate the product of likelihood function L(θ) and prior probability π(θ) over the entire prior parameter space. In Bayesian inference, the posterior probability distribution of the model parameters is normalized by the constant Bayesian evidence Z and can be expressed as:

(12) $$p\left(\theta \right)=\frac{L(\theta )\pi (\theta )}{Z}.$$

The denominator in Equation (12) is known as the Bayesian evidence and is utilized for model comparison to choose the best model or probability distributions where the parameters have to be estimated. The Bayesian evidence Z in this study for the residuals *W* and models M(θ) with parameters θ is given by:

(13) Z=p(W|M(θ))=∫L(θ)·π(M(θ))dθ.

**Figure 3:**
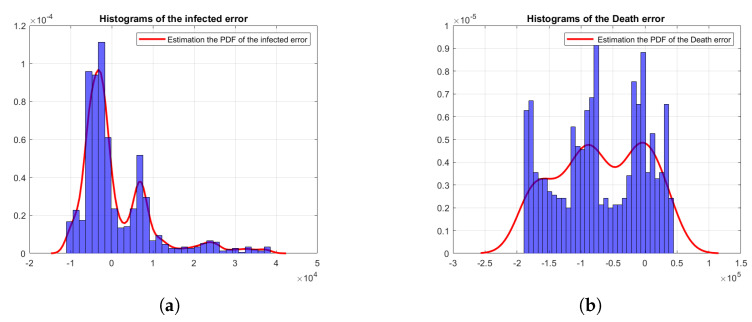
Histogram of the infected and death errors of the output of Equation (3). (**a**) Histogram of the $w_{I}$; (**b**) histogram of the $w_{D}$.

The main advantage of nested sampling can perform well with high dimensional integration, which is sampled from the prior range towards higher likelihood regions as well as from the multimodal likelihood shape [37]. Once the estimates of the measurement error of Equation (3) are computed by the aforementioned distributions, we will be able to produce the posterior samples and the marginal likelihood Z for each of them. The number of sampled points and the range of the uninformative prior are shown explicitly with numerical values in Section 4.2. Figure 3a,b show the frequency of the infected error and the frequency of the death error, which are the differences between the deterministic model fit and the real data, where the distributions are constructed by the kernel density estimate (KDE). The number of bins for both the infected and death error data has been selected using the square root of the number of reported data points, which we also use while calculating the likelihood function. Hence, for accurate characterization of the errors, we need to estimate the parameters of the probability distributions. The Bayesian evidence calculated from the nested sampling helps in selecting the right model for the error distribution, which in turn leads to more accurate state estimation using the EKF. Additionally, the nested sampling provides the posterior samples to estimate the covariance matrices for modelling the correlations between the two error variables, i.e., the infected and death errors.

## 4. Results and Discussions

### 4.1. Bayesian Inference Results and Error Distributions

**Figure 4:**
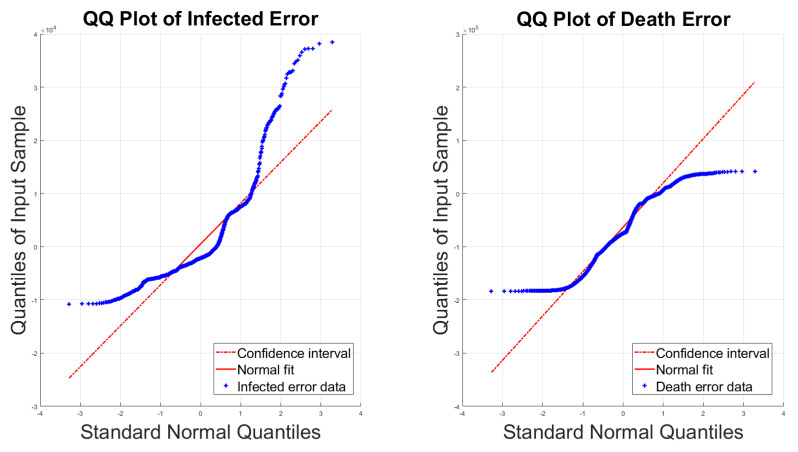
Quantile–Quantile plot of COVID-19 error predictions for infected and death residuals.

In the Bayesian inference framework, choosing the appropriate prior range is not a trivial task in the absence of expert knowledge of the range of possible values of each unknown parameter. In this context, we assumed the uninformative prior of the underlying parameters of the noise distributions to be uniform distributions with specified bounds. We considered the models that assume the quantity of interest is symmetrically and asymmetrically distributed. Since skewness is the key parameter in the skewed distributions, choosing the range of the skewness parameter should be selected carefully in order to obtain the right posterior inference. A discussion about the sensitivity of prior distribution in the asymmetric distributions can be found in [38,39,40]. We examine the skewness index of the residuals of the infected and death cases by using the sample coefficient of skewness, which is an unbiased estimator for the third central moment obtained by dividing the third central moment over the cubed standard deviation as follows:

(14) $$\mathrm{skewness}=E [{\left(y-\mu \right)}^{3}]/\sigma^{3}$$

where μ presents the sample mean and the σ is the sample standard deviation [41,42]. The skewness values calculated using Formula (14) are $w_{I}=1.84$ and $w_{D}=-0.1325$, which indicate that the residual data have departed from normality. Even with the small deviations from normality in death cases where all cases are different from 0, it is significant to be considered, as pointed out in [43]. Selecting the skewness range has been debated in much of the statistics literature; e.g., Ref. [44] describes that if the skewness is greater than 1 in the absolute value, then the data are highly skewed, while skewness between 0.5 and 1 is moderately skewed and skewness between 0 and 0.5 is fairly symmetrical. In addition, Ref. [45] suggested the range of skewness is ±1.5 and ±2, as also described in [46]. To evaluate the magnitude of skewness of the quantity of interest (the residuals), Figure 4 detects the skewness between samples fitted by the skew-normal distribution versus those fitted by a Gaussian distribution using a QQ-plot. Subsequently, from the distributional view of the residual data in Figure 4, the dispersion from normality is clearly identified. However, the prior boundaries in this study are chosen based on the associated data, shown in Table 1, where the prior assigns the same range across different models to make the comparison fair and consistent. Furthermore, two different model comparison approaches are illustrated in the next Section 4.2.

### 4.2. Assumptions on the Error Distributions

In this section, we aim to calculate the evidence of the aforementioned distributions with four different models:

- The MVN distribution with a full covariance matrix Σ between *I* and *D*;
- The MVN distribution with no correlation between *I* and *D*, i.e., diagonal covariance matrix Σ;
- The MSN distribution with a full covariance matrix, Σ, between *I* and *D*;
- The MSN distribution with no correlation between *I* and *D*, i.e., diagonal covariance matrix, Σ.

Conventionally, the first assumption is denoted by Model 1, the second assumption by Model 2, the third assumption by Model 3 and the last assumption by Model 4.

### 4.3. Sampling from the Posterior of the Unknown Noise Parameters Using Nested Sampling

A set of samples are drawn from the prior distributions for the above statistical models and the prior range is decreased iteratively as the live points navigate towards higher likelihood regions. Thus, during each iteration, the lowest likelihood value is identified and replaced with a new point randomly drawn from the prior distribution subject to the constraint that the newly sampled point must have a likelihood greater than the discarded point. This process continues until the difference in the evidence calculated from the samples gathered by all live points become smaller than a specified threshold (Δlog(Z)<0.001). At this point, the nested sampling algorithm terminates and returns the final estimate of the log-evidence log(Z), along with its confidence interval. The posterior distribution of the unknown noise parameters is calculated as a by-product of the evidence calculation process. The posterior represents the multivariate probability distribution of the unknown model parameters, given the observed data, and can be used for model comparison, prediction and inference.

**Figure 5:**
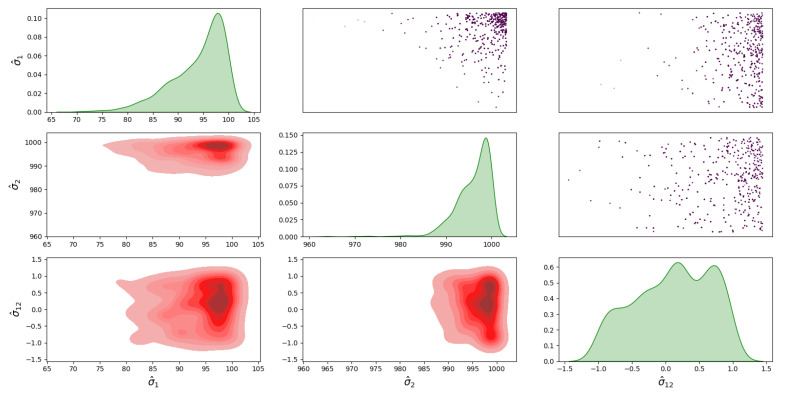
Posterior distribution of Model 1 for the infected and death errors with the correlated covariance matrix.

**Figure 6:**
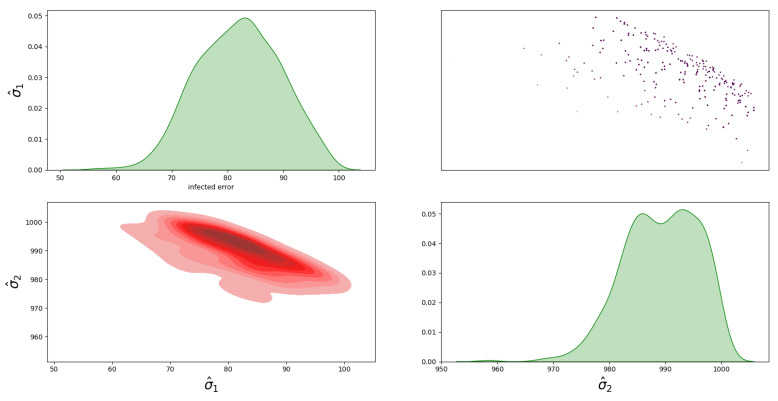
Posterior distribution of Model 2 for the infected and death errors with no correlation in the covariance matrix.

**Figure 7:**
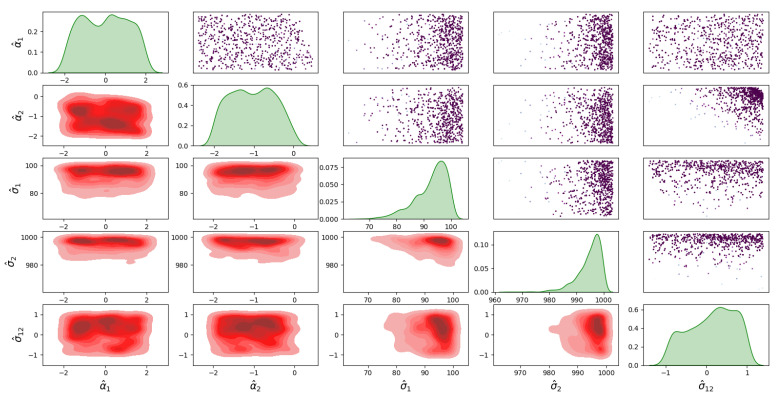
Posterior distribution of Model 3 for the infected and death errors with correlation in the covariance matrix.

**Figure 8:**
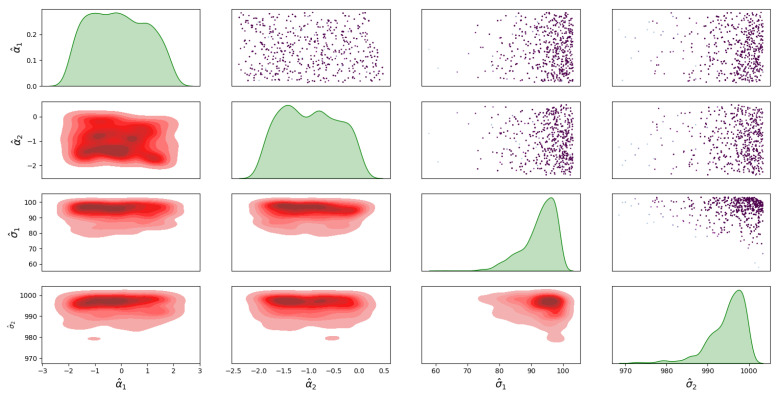
Posterior distribution of Model 4 for the infected and death errors with no correlation in the covariance matrix.

For Model 1, the posterior distribution is shown in Figure 5, which shows the correlated samples under the MVN distribution. The main diagonal in Figure 5 shows the 1D marginal likelihood and provides the maximum a posteriori (MAP) estimate for the parameters ${\hat{\sigma }}_{1}$, ${\hat{\sigma }}_{2}$ and ${\hat{\sigma }}_{12}$. The upper triangular part in Figure 5 shows the scatter plots of the parameters coloured at each point by the log-likelihood values to analyse the correlation between the parameters. Moreover, the lower triangular part shows the marginal bivariate posterior distributions in 2D, estimated by the Gaussian KDE. For the posterior distribution of Model 2, the covariance matrix is diagonal under the MVN distribution is shown in Figure 6. For the MSN distribution, the posterior of Model 3 is shown in Figure 7, and the posterior of Model 4 is shown in Figure 8. Furthermore, Figure 5, Figure 6, Figure 7 and Figure 8 all have similar interpretations in terms of the upper/lower diagonal parts with additional skewness parameters ${\hat{\alpha }}_{1}$ and ${\hat{\alpha }}_{2}$ in the MSN distribution cases.

### 4.4. Bayesian Model Comparison for Selecting the Best Noise Distribution

Table 2 shows the summary of the number of live points ($N_{\mathrm{live}}$), which is a set of points in the prior range which are used to calculate the evidence while navigating towards the high likelihood regions and explore the shrinking prior space efficiently, along with the total number of the likelihood function evaluations ($N_{\mathrm{like}}$) and the log-evidence (logZ) for each chosen noise distribution obtained by run MultiNest sampling [47] from the multimodal likelihood for all the generated samples. The average number of the $N_{\mathrm{live}}$ iterations is proportional to the number of dimensions of the corresponding model, and in this study, we chose $N_{\mathrm{live}}=20\times$ dimensions for the unknown PDF parameters. The $N_{\mathrm{live}}$ can be user-defined to make a trade-off between the computational cost and the accuracy of the calculated evidence and posterior distribution. The tolerance between successive iterations is chosen as Δlog(Z)<0.001. A higher logZ value is associated with the best-fitted distribution, which is the model MVN with correlation, followed by the MSN with correlation, which reflects a linear relationship between the infected sample and the death sample. Despite the tiny difference in the logZ values between Model 1 and Model 3, we can justify that the MVN model is a better choice over the MVN model since the skewness index is fairly large for death cases and can be in an approximately normal scope for our data. However, for highly skewed data, it is likely that the statistical model selection based on the associated logZ values will tend towards the asymmetric distributions. Now, we calculate the mean posterior of these fitted statistical models in the measurement covariance matrix Ω of the EKF in order to optimize the state estimates of the hidden or unmeasured variables, which has been shown in the Section 4.5.

The comparison between two different models can be evaluated using the Bayes factor, as suggested in the literature, e.g., [48,49,50], to measure the relative strength of evidence for one model over another. The Bayes factor ρ provides the posterior odds, which is the ratio of the marginal likelihoods multiplied by the model prior P(Model) given by the observed data *W*. Jeffrey’s scale [51] provides a qualitative interpretation of the Bayes factor values that can be used to assess the strength of the evidence of one model with respect to another model. Thus, the degree or strength of evidence can be determined by Jeffrey’s scale. It suggests an index to distinguish between the models, with 1<ρ<2.5 being substantial, 2.5<ρ<5 being strong and ρ>5 being decisive, as reported in [32]. According to Table 2, the higher logZ is associated with Model 1, then computing the Bayes factor for this model versus other models can be presented as:

(15) $$\rho_{1}=\log [\frac{P\left(\mathrm{Model}-1|W\right)}{P\left(\mathrm{Model}-2|W\right)}]=\log\frac{P\left(W|\mathrm{Model}-1\right)\cdot P\left(\mathrm{Model}-1\right)}{P\left(W|\mathrm{Model}-2\right)\cdot P\left(\mathrm{Model}-2\right)}=\log [\frac{Z_{\mathrm{Model}-1}}{Z_{\mathrm{Model}-2}}]$$

$$=\log [Z_{\mathrm{Model}-1}]-\log [Z_{\mathrm{Model}-2}]=3.7253,\;\mathrm{strong}\;\mathrm{evidence}.$$

This provides evidence that the data are more likely to be under Model 1 than Model 2. Now,

(16) $$\rho_{2}=\log [\frac{P\left(\mathrm{Model}-1|W\right)}{P\left(\mathrm{Model}-3|W\right)}]==\log\frac{P\left(W|\mathrm{Model}-1\right)\cdot P\left(\mathrm{Model}-1\right)}{P\left(W|\mathrm{Model}-3\right)\cdot P\left(\mathrm{Model}-3\right)}=\log [\frac{Z_{\mathrm{Model}-1}}{Z_{\mathrm{Model}-3}}]$$

$$=\log [Z_{\mathrm{Model}-1}]-\log [Z_{\mathrm{Model}-3}]=0.331,\;\mathrm{weak}\;\mathrm{evidence}.$$

In addition, for Model 4, we obtain:

(17) $$\rho_{3}=\log [\frac{P\left(\mathrm{Model}-1|W\right)}{P\left(\mathrm{Model}-4|W\right)}]==\log\frac{P\left(W|\mathrm{Model}-1\right)\cdot P\left(\mathrm{Model}-1\right)}{P\left(W|\mathrm{Model}-4\right)\cdot P\left(\mathrm{Model}-4\right)}=\log [\frac{Z_{\mathrm{Model}-1}}{Z_{\mathrm{Model}-4}}]$$

$$=\log [Z_{\mathrm{Model}-1}]-\log [Z_{\mathrm{Model}-4}]=0.333,\;\mathrm{weak}\;\mathrm{evidence}.$$

From the comparative results obtained from $\rho_{2}$ and $\rho_{3}$, it suggests that Model 3 and Model 4 are equally likely, since their logZ values are close to each other.

### 4.5. State Estimation Using Extended Kalman Filters with the Estimated Noise Distributions

The aim of this subsection is to show the fine-tuning of the EKF with different measurement error covariances Ω and distributions given from the mean posterior of the samples, as shown in Table 3, as compared to the value in the last row, i.e., the EKF5, which is a pre-assumed noise covariance reported in [1]. The root mean squared error (RMSE) for each case between the performance of EKFs and the reported data is presented using:

(18) $$\begin{array}{cc} \mathrm{RMSE} & =\sqrt{\frac{1}{n}\sum_{i=1}^{n}{\left(X_{\mathrm{reported}\;I,D}-X_{\mathrm{EKFs},I,D}\right)}^{2}},\\ \; & =\sqrt{\frac{1}{n}\sum_{i=1}^{n}e_{i}^{2}}. \end{array}$$

**Figure 9:**
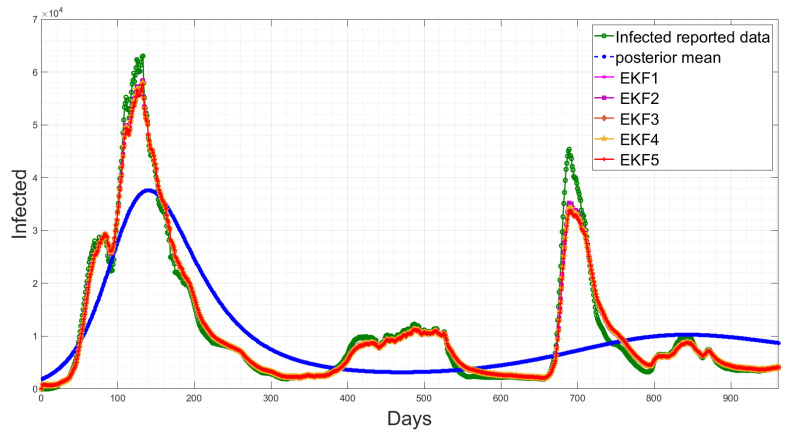
Temporal comparison of the EKFs’ performances for the active cases along with the reported data and posterior mean response.

**Figure 10:**
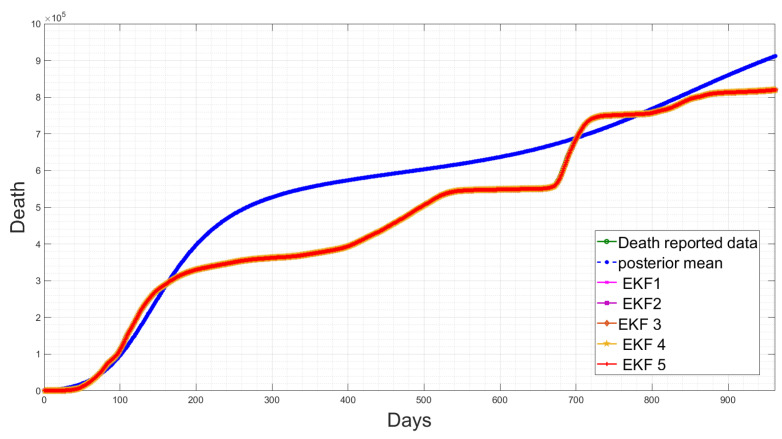
Temporal comparison of the EKFs’ performances for the death cases along with the reported data and posterior mean response.

**Figure 11:**
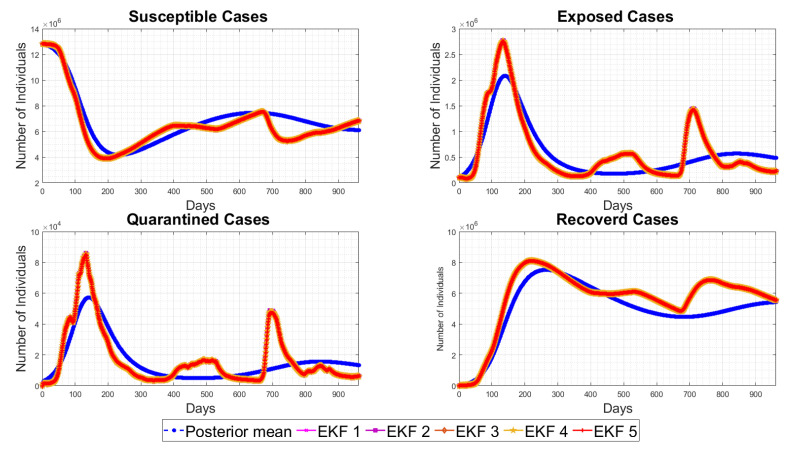
EKFs’ performances for the unobserved hidden states, i.e., Susceptible, Exposed, Quarantined, Recovered cases, as compared to the posterior mean response.

**Figure 12:**
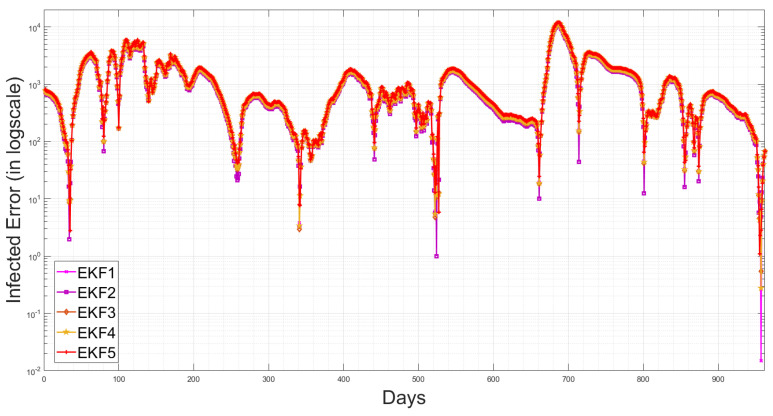
Semi-log plot of the infected cases for the proposed EKFs.

**Figure 13:**
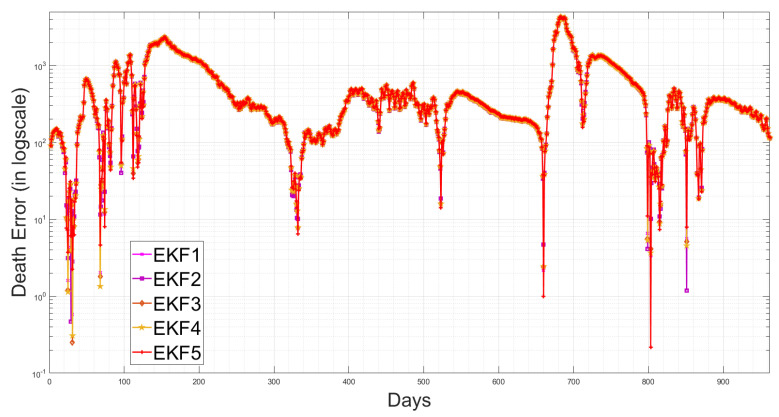
Semi-log plot of the death cases for the proposed EKFs.

Table 3 shows that the EKF2 using MVN with no correlation has a lower RMSE, as compared to the other EKF variant-based RMSE values. This indicates that the EKF2 predictions are closer to the actual values and have the best accuracy among those models. Furthermore, from Table 3, we observe that EKF1, EKF2, EKF3 and EKF4 all are able to reduce the prediction errors as compared to EKF5, which uses the pre-assumed covariance reported in [1], for the infected cases, while there are slight differences in the death cases. Consequently, using more accurate noise covariance in the stochastic model will enhance the EKF’s prediction performance and provide further optimal results in EKF-based state estimation. Furthermore, our methodology focuses on noise model validation using Bayesian evidence and the RMSE score between the predicted and reported infected/death cases. Fitting the deterministic component of the EKF was reported previously in [1], and here, we report the estimated parameters with longer-term historic data. The RMSE has been used as a standard metric to quantify the prediction results in pandemic modelling. However, it is worth mentioning that all the results shown in this paper are subjected to the structure of the underlying nonlinear differential equation, i.e., the structure of the chosen SEIQRD model with reinfection. Since there are several epidemic models reported in the literature with different levels of complexity, if the deterministic model is changed to fit the raw data and generate residuals while including more compartments, geospatial distributions or age/social groups, the ranking of the EKF’s performance may be different than the results reported in this paper. The simulations of EKFs have been performed for the number of active cases, as shown in Figure 9, and for the death cases in Figure 10, along with the four hidden states (Susceptible–Exposed–Recovered–Quarantined) in Figure 11. Moreover, we visualize the differences between the individual EKF’s performance in the semi-log plot for the infected cases in Figure 12 and for the death cases in Figure 13.

The mean posterior estimates of the SEIQRD model parameters for the long-term Saudi Arabia COVID-19 data are reported in Table 4, which is an improved version over [1] with the most updated data.

## 5. Conclusions

This paper presents a new method for modelling the uncertainty in the epidemiological SEIQRD model considering dependence and independence in the observation noise. The method is based on the Bayesian inference method called nested sampling, and we provide an extensive analysis to quantify the uncertainty of the noise distributions and feed it back into the Kalman filtering framework for state estimation. We examined the full covariance as well as the diagonal case for each model since it is unknown which model is the best for state estimation. We have shown through simulation studies a comparison between the multivariate normal distribution and the multivariate skew normal distributions for the observation error. We have compared our method using five alternative formulations of the EKF with different noise distributions and manually tuned noise covariance matrices in order to make a fair comparison between different methods. Our main objective here is to obtain accurate state estimation for unmeasurable or hidden states in the epidemiological model and not just forecasting measurements, unlike black-box, non-parametric time series models.

### 5.1. Main Contributions and Limitations

The main contribution of the proposed approach is to fine-tune the EKFs, including the nonlinear epidemiological SEIQRD model with reinfection, using different structures of the measurement error distribution and covariance Ω in a more systematic way as compared to the previous work in [1]. The previous work in [1] assumed that Ω can take an arbitrary value and can be manually tuned, and despite that, we obtained decent prediction performance using the EKF. In this paper, we further fine-tune all the noise parameters in EKF based on the Bayesian evidence criterion and we conclude that all the examined four noise distributions are better than the pre-assumed or hand-tuned noise covariance, based on the RMSE criterion of the predictions of two measured variables-infected and death cases. Moreover, this generalized method can be performed regardless of which type of EKF is used including being agnostic on the chosen deterministic compartmental model structure. The second major contribution of this paper is to estimate the hidden states of COVID-19 dynamics from the chosen SEIQRD model, i.e., the susceptible, exposed quarantine and recovered cases, along with their respective uncertainty bounds, which is a non-trivial task, using standard time-series models. Such estimation is crucial to supporting decision making for pandemic management by the government. However, in this study, we do not tune the system covariance Ξ due to the high computational burden since our goal is to match the realistic measurement error in *I* and *D*.

There have been numerous studies on pandemic modelling based on a single country or even smaller regional data in different states or counties. However, parameter estimation, comparing the deterministic and stochastic parts of models and designing state estimation algorithms using nonlinear models are not straightforward tasks. Therefore, our study was based on only Saudi Arabian data, and we plan to extend it to other countries in future works. Although many countries have published COVID-19 data, mostly their cumulative death records, data on actives cases (different from daily new cases and confirmed and cumulative infected data) which is traditionally used in pandemic models are very hard to obtain. Moreover, the range of estimated parameters changes with the infrastructure of different countries, which motivated us to develop the methodology for a single country initially and extend it in the future by modifying the prior range. However, for better reproducibility, we report the estimated SEIQRD model parameters in Table 4 before noise modelling in the EKF algorithm.

### 5.2. Future Work

Several extensions of the reported work are possible:

- This work can be further extended with other, more complex nonlinear epidemiological compartmental models under the EKF framework with more experimental evidence.
- Another possible future direction is to include more model comparisons within different skewness distribution families using similar approaches such as the class of skewness distributions presented in [52]. However, the challenge with this class is dealing with the extra parameters as well as prior range selection, which should be carried out carefully as there is a lack of interpretability in the literature which needs further investigation.

Overall, there is still a risk of a global pandemic like the one experienced during COVID-19, which is assumed in the SEIQRD model, and adopting various protective measures to prevent the transmission can significantly minimise that risk. It is fundamental to stay informed using recent scientific research and to continue to follow the guidance of public health organizations in order to protect ourselves and others. We are currently in the stage of living with COVID-19 after the substantial effort of developing successful treatments and effective vaccines, although there may be different variants of the virus that have not been discovered yet. Forecasting and developing predictive state estimation strategies for potential future pandemics is a challenging task for any government and has not been discussed well in the contemporary scientific literature since it requires interdisciplinary research involving epidemiology, public health, medicine, applied mathematics and statistics. In [53], the authors discussed some future pandemic threats associated with changes in the biosphere, such as climate change, loss of biodiversity and deforestation. However, this pandemic made the world vigilant and more prepared for other global crises. Consequently, we can analyse other potential future pandemics which are related to infectious diseases and pathogens that could threaten public health. Investigation into possible future pandemics may be classified into a few categories as follows:

- Unknown microbes or microorganisms that arrive from space, which are called panspermia, as discussed in [54,55]; however, there is a lack of evidence for this hypothesis and microbial data collection in space is limited due to the high cost, the complexity of the experimental setup and the high level of risk involved. As there are no clear findings of microorganisms coming from asteroids, comets or spacecraft, the evidence of possible infection is rather low. COVID-19 data can help in improving modelling of unknown pandemics from outer space.
- The outbreak of experimental microbes in scientific laboratories, due to accidents or poor management practices, can result in infections such as Brucella abortus, which can cause foetal death in pregnant women. Moreover, the origin of COVID-19 is still unknown, and whether it emerged through natural spillover, trans-species migration or a laboratory accident is still uncertain. However, a laboratory accident cannot be excluded as a potential risk for similar or even larger future pandemics, as discussed in [56,57].
- Future pandemics may also be caused by anthropogenic roots such as political conflicts or wars between countries, continents and specific genotypes, which can be modelled and controlled using the COVID-19 data as a test scenario [58,59]. This may also include manipulating the birth rate and other constants between the model compartments related to the health system’s infrastructure, such as the hospital capacity, quarantine period and reinfection rate in a country or region.

We have learnt a lesson from the COVID-19 pandemic, which had the largest number of deaths in recent history. We developed robust combat policies and controlled public fear and uncertainty affecting quality of life, the economy and education to name a few amongst many social aspects [60]. Most modern epidemiological studies in humans, animals and plants utilize a wide range of mechanistic compartmental models as well as real-world data to accurately characterize the evolution of an epidemic through estimation algorithms and to assess risk levels for possible future pandemics which may cause wider-scale disruptions in daily life. Therefore, our modelling and state estimation results using the Saudi Arabian COVID-19 data can be seen as a generalized framework for systematically estimating the deterministic and stochastic components of a pandemic within an extended Kalman filtering framework utilizing the SEIQRD and even more complex compartmental models.

## Figures and Tables

**Table 1:** The uninformative prior distribution for the proposed models.

Model	${\hat{\sigma }}_{1}$	${\hat{\sigma }}_{2}$	${\hat{\sigma }}_{12}$	${\hat{\alpha }}_{1}$	${\hat{\alpha }}_{2}$
MVN with correlation	$U [100,1000]$	$U [100,1000]$	U[−1,1]	- ${}^{†}$	-${\;}^{†}$
MVN without correlation	$U [100,1000]$	U[100,1000]	-${\;}^{†}$	- ${}^{†}$	- ${}^{†}$
MSN with correlation	$U [100,1000]$	$U [100,1000]$	U[−1,1]	U[−2,2]	U[−2,2]
MSN without correlation	$U [100,1000]$	U[100,1000]	- ${}^{†}$	U[−2,2]	U[−2,2]

${}^{†}$ does not exist for the given distribution.

**Table 2:** Bayesian evidence for each error distribution along with the number of live points $N_{\mathrm{live}}$ and the number of likelihood function evaluations $N_{\mathrm{like}}$.

Model	$N_{\mathrm{live}}$	logZ	$N_{\mathrm{like}}$
Model-1: MVN with correlation	60	−263.215 ± 0.329	715
Model-2: MVN without correlation	40	−266.940 ± 0.403	463
Model-3: MSN with correlation	100	−263.546 ± 0.265	1239
Model-4: MSN without correlation	80	−263.548 ± 0.297	989

**Table 3:** Root Mean Square Errors (RMSEs) of the proposed EKFs with different noise distributions and covariance matrices.

EKF	Covariance Matrices	Infected RMSE	Death RMSE
EKF1 MVN with correlation	Ξ = 500 × $I_{6\times 6}$, Ω = $[\begin{array}{cc} 93.81 & 0.1001\\ 0.1001 & 995.91 \end{array}]$	67.1907	26.9871
EKF2 MVN without correlation	Ξ = 500 × $I_{6\times 6}$, Ω = diag[82.017, 989.20]	61.2482	26.6587
EKF3 MSN with correlation	Ξ = 500 × $I_{6\times 6}$, Ω = $[\begin{array}{cc} 92.53 & 0.13469\\ 0.13469 & 994.59 \end{array}]$	66.5684	26.9649
EKF4 MSN without correlation	Ξ = 500 × $I_{6\times 6}$, Ω = diag[93.17, 994.56]	66.8042	26.9032
EKF5	Ξ = 500 × $I_{6\times 6}$, Ω = diag[100, 1000], Ref. [1]	70.1258	27.0861

**Table 4:** Mean Posterior estimates of the SEIQRD model parameters for the long-term Saudi Arabia COVID-19 Data.

Parameter	Parameter Value		
	Description	Mean	Standard Deviation
β	infection rate	3 $\times 10^{-8}$	4.4 $\times \;10^{-9}$
α	reinfection rate	0.0028	1.4 $\times \;10^{-5}$
ϵ	incubation period	0.0353	0.00361
*q*	quarantine rate	0.9593	0.0033
qt	quarantine period	0.5939	0.0521
*d*	death rate	4 $\times \;10^{-3}$	2.7 $\times \;10^{-5}$
γ	recovery rate	0.9586	0.0031

## Data Availability

The data are available from the lead author upon reasonable requests.

## Abbreviations

The following abbreviations are used in this manuscript:

KF	Kalman filter
EKF	Extended Kalman filter
MVN	Multivariate normal distribution
MSN	Multivariate skew normal distribution
PDF	Probability density function
CDF	Cumulative density function
KDE	Kernel density estimate
MAP	Maximum a posteriori estimate
RMSE	Root Mean Squared Error
